# A taxonomic dataset of preserved specimen occurrences of *Theobroma* and *Herrania* (Malvaceae, Byttnerioideae) stored in 2020

**DOI:** 10.3897/BDJ.11.e99646

**Published:** 2023-03-30

**Authors:** Matheus Colli-Silva, James E. Richardson, José R. Pirani

**Affiliations:** 1 Department of Botany, Institute of Biosciences, University of São Paulo, São Paulo, Brazil Department of Botany, Institute of Biosciences, University of São Paulo São Paulo Brazil; 2 School of Biological, Earth and Environmental Sciences, University College Cork, Cork, Ireland School of Biological, Earth and Environmental Sciences, University College Cork Cork Ireland; 3 Tropical Diversity Section, Royal Botanic Garden Edinburgh, Edinburgh, United Kingdom Tropical Diversity Section, Royal Botanic Garden Edinburgh Edinburgh United Kingdom; 4 Faculty of Natural Sciences, Rosario University, Bogotá, Colombia Faculty of Natural Sciences, Rosario University Bogotá Colombia

**Keywords:** Amazonia, chocolate, flowering plants, herbarium collections, online repository

## Abstract

**Background:**

Species from the "cacao group" are traditionally allocated into two genera, *Theobroma* and *Herrania* (Malvaceae, Byttnerioideae), both groups of Neotropical species economically relevant, such as the cacao tree (*Theobromacacao*), which forms the source of chocolate. This study aimed at compiling and describing a dataset of preserved specimen collections available in the Global Biodiversity Information Facility repository (GBIF) for Tropical Americas. Data were exhaustively revisited and analysed in terms of taxonomic identity, conditions of collection and georeferencing, all of which should enable downstream taxonomic, geographic and evolutionary analyses.

**New information:**

Our dataset compiles 7975 records of preserved specimen collections found at herbaria. Records are from 18 species of *Theobroma* and 14 of *Herrania*, occurring in 60 countries or major territories, with two species endemic to a single country (*H.kofanorum* from Ecuador and *H.laciniifolium* from Colombia). Occurrence records are mostly restricted to the Amazon rainforest and species with more occurrence records are *cupuí*, *T.subincanum* (1535 records), followed by the cacao tree, *T.cacao* (1500 records), the latter having cultivated specimens in Africa, Asia and Oceania. In the case of the genus *Herrania*, *H.nitida* and *H.purpurea* are the species with the majority of occurrences (respectively, 431 and 273 records). Most of the botanical samples from these genera are found in American, Brazilian and Colombian collections, with a particular strength for American herbaria. We describe how occurrence records are spread spatially and temporally and highlight key field expeditions responsible for enhancing most of the knowledge of cacao and its wild relatives, especially in countries where they prevail, such as Colombia (with 29 species), Ecuador (23 species), Brazil (18 species) and Peru (15 species). Specifically, expeditions in these countries were led by American and European initiatives in conjunction with local funding in the mid-20^th^ century. We emphasise how initiatives of such kind seems to have weakened in the 21^st^ century and most of the collections of *Theobroma* and *Herrania* made afterwards are from various collectors that seek to resample specimens in already explored sites.

## Introduction

As holders of most of vascular plant species richness in Earth (Ulloa Ulloa et al. 2017), biodiversity documentation represents an enormous challenge for Tropical Americas' emerging countries, especially in areas that associate high diversity with low collecting efforts, such as in the Amazon rainforest (Daly and Prance 1989, Schulman et al. 2007). This is the case of species from the genera *Theobroma* L. and *Herrania* Goudot, members of the mallow and the cacao family (Malvaceae), an important component of tropical vegetations worldwide. *Theobroma* and *Herrania* are closely-related genera and both groups are marked by their baciform fruits with a sweet pulp eaten by humans and monkeys (Bletter and Daly 2009).

The last comprehensive contributions on the diversity of the cacao group are the revision of *Theobroma* (Cuatrecasas 1964) and the synopsis of *Herrania* (Schultes 1958). Both studies have provided one of the yet few attempts to properly describe a total of 39 species for the two genera, recognising 22 species for *Theobroma* and 17 for *Herrania* in their circumscription. No taxonomic revisions have been conducted since then.

**Figure 1.**
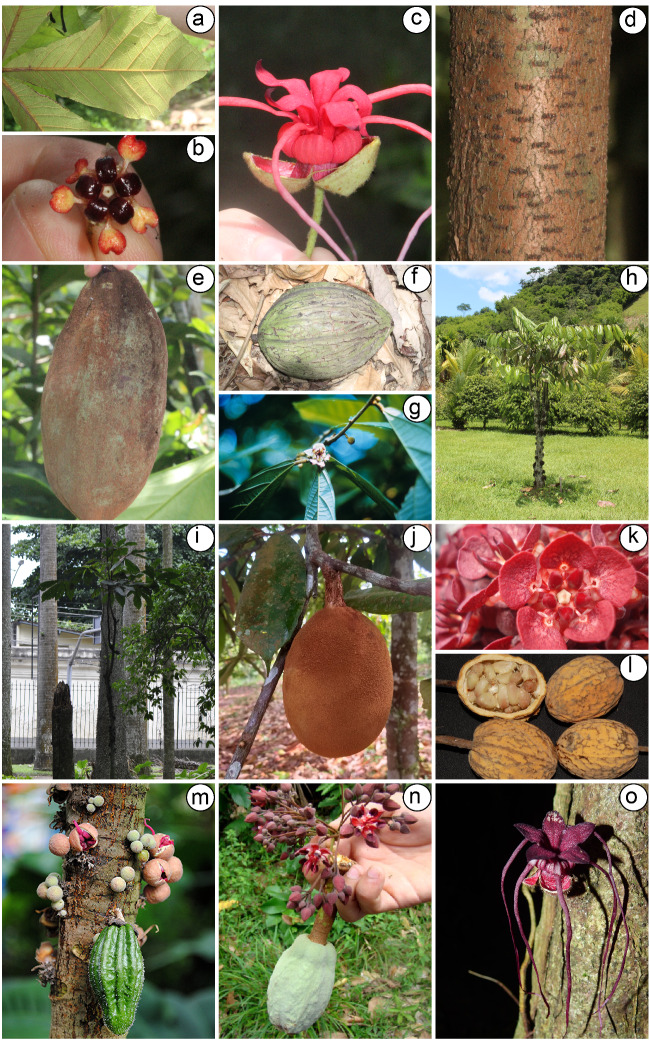
General morphology of *Theobroma* L. and *Herrania* Goudot. **a** leaves of *H.mariae* Goudot, focusing on one leaflet; **b** flower of *T.obovatum* Klotzsch ex Bernoulli; **c** flower of *H.pulcherrima* Goudot; **d** bark of *T.obovatum*, notice the marked presence of lenticels; **e** fruit of *T.angustifolium* DC.; (f) fruit of *T.bicolor* Humb. & Bonpl.; **g** flowering branch of *T.grandiflorum* (Willd. ex Spreng.) K.Schum.; **h** general aspect of a small individual of *T.speciosum* Willd. ex Spreng.; **i** general aspect of *H.nitida* (Poepp.) R.E.Schult.; **j** fruit of *T.grandiflorum*; **k** flowers and **i** fruits of *T.speciosum*; **m** main stem of *H.purpurea* (Pittier) R.E.Schult. with flowers and fruits growing on the trunk; **n** reproductive structures of *T.glaucum* H.Karst.; **o** flower of *H.kanukuensis* R.E.Schult. Photos: M. Pellegrini (a-f, h, i); J.E. Richardson (k-n); R.A. Howard (g), obtained from iNaturalist; R. Chapalbay (j), obtained from iNaturalist; S. Sant (o), obtained from iNaturalist. All photos are under CC BY-NC 4.0 license.

Morphologically, *Herrania* is distinguished from *Theobroma* by its branching architecture (monopodial vs. sympodial in *Theobroma*), compound leaves (vs. simple leaves in *Theobroma*), as well as by the trimerous calyx (vs. usually pentamerous in *Theobroma*) and for having the upper portion of an unguiculate petal (the ligule) much longer in *Herrania* than in *Theobroma* (Schultes 1958, Cuatrecasas 1964, Daly and Prance 1989) (Fig. 1c). In fact, Herrania is sometimes considered as a subgenus of Theobroma for other authors (Schumann 1886, Ducke 1940), but differences in leaves, flower morphology and even in the fruits are relevant features that currently separate these entities as two genera apart (Cuatrecasas 1964, Schulman et al. 2007) (Fig. 1).

Perhaps due to its long historical and economical importance, wild cacao species are well-known by many American societies. Most species are locally known as *cacao*, *cacao-del-monte, cacaorana*, *cacauí, cupuí, sasha-cacahuillo* or derivatives and *Herrania*, despite being relatively less known than its sister-genus *Theobroma*, can be rapidly recognised as a cacao relative and is locally called as *cacau-jacaré* or *cacao-azul* (blue cacao). One particular species, *Theobromacacao* L., forms the source of chocolate and it is potentially native to Western Amazonia, but widely cultivated in many areas in Mesoamerica and overseas (see, amongst other references, Zarrillo et al. (2018), Fouet et al. (2022)).

Field expeditions in the Amazon Basin in search for wild cacao species were carried out in the 20^th^ century, alongside the rise of the chocolate industry and the development of Brazil, Peru and Colombia towards inner areas. The Anglo-Colombian Cacao Collecting Expedition (Baker et al. 1953) and further expeditions maintained by the *Projeto Flora Amazônica* in Brazil (Prance et al. 1984) contributed with the increase of wild cacao collections at the time. However, as early as the 17^th^ century, some names highlight, such as José Celestino Bruno Mutis y Bosio (1732-1808), a Spanish botanist who led a long expedition in Nova Granada (currently Colombia, Ecuador, Panama and Venezuela), when many samples of *Theobroma* and *Herrania* were collected. Another important mention is Francisco Jose de Caldas (1768-1816), who made the first cacao transects mapping cacao regions from Bogotá (Colombia) up to Quito (Ecuador), mostly in 1803 (González-Orozco et al. 2015, González‐Orozco et al. 2021).

These expeditions enabled the development of subsequent taxonomic treatments for the groups mentioned above (Schultes 1958, Cuatrecasas 1964). To overcome such challenges, endeavours in making existent collections more accessible for data consuming and mobilisation have increased (Pyke and Ehrlich 2010, Nualart et al. 2017), enabling rapid, but not less efficient synthesis studies on the known and unknown biodiversity. This is allied with the arise of biodiversity data repositories that gather information from the most disparate sources, namely the Global Biodiversity Information Facility (GBIF; Robertson et al. (2014)), the largest repository of its kind. Additionally, further datasets that gather historical publications (BHL, the Biodiversity Heritage Library, https://www.biodiversitylibrary.org/) or scientific names with protologue information (IPNI, the International Plant Names Index, https://www.ipni.org/) and floral monographs (BFG 2021) unify a once fragmented knowledge which is now integrable.

## General description

### Purpose

We aimed at building a dataset of preserved specimen records of cacao and its wild relatives (genera *Theobroma* and *Herrania*), with a particular strength in Tropical Americas, where both genera are native to, but eventually also comprising records overseas. This dataset includes revisited data only of preserved specimen collections (i.e. data deposited in herbaria) and should enable downstream works with systematics, conservation and evolution of a Neotropical group of relevance in Tropical Americas.

### Additional information

Our dataset was first obtained from the GBIF database, downloaded on 3 August 2020 (GBIF.org 2020). This initial dataset has 15849 entries from 313 datasets, including thirteen entries of fossil specimens, 919 entries of human observations, 287 entries of living specimens, 28 entries of machine observations, 81 entries of material samples (e.g. records from spirit collections), 11305 entries from preserved specimen collections (i.e. materials found at herbaria) and 3216 entries of unknown precedence. It should be noted that, for the purposes of this study, only preserved specimen collections were considered, because these can be reached at herbarium collections and be properly attested with respect to their geographic origin and taxonomic identity. For these, herbarium acronyms for preserved specimen collections followed Thiers (2021) designations.

The downloaded dataset (GBIF.org 2020) was the gold-standard source for an extensive taxonomic revision conducted by the authors of this study. This revision included both field expeditions, as well as the study of the preserved specimen materials, morphological and phylogenetic analyses which will ultimately derive in the publication of a new, updated taxonomic revision for the taxa being studied in here. After data manipulation, data cleaning and checking coordinates and the precedence of the vouchers, we kept 7975 preserved specimen records for 32 species in two genera. GBIF-mobilised data are available as Supplementary Material (Suppl. material 1).

## Geographic coverage

### Description

Georeferencing followed standard protocols described in Magdalena et al. (2018). As only a small proportion of records of Amazonian collections are georeferenced and auto georeferencing in Amazonia is a difficult task (Hopkins 2019), we worked to provide the best source of available geographical information, based on exhaustive attempts at estimating the best locality for each voucher. Additionally, our dataset was subject to an automated locality standardisation through functions provided in the “plantR” v. 0.1.5 package in R Environment (R Core Team 2020, Lima et al. 2021).

A total of 5277 entries (66%) maintained their coordinates as informed in the voucher label, while 1960 entries (25%) had dubious or ambiguous coordinates and could not have a locality properly assigned (Table 1). Cases such as inaccurate records referred to vouchers whose coordinates were all indiscriminately approximated to country centroids (as is the case of many collections from F, MO and US collections) fell into this category, for example. Still, 738 entries (9%) were georeferenced accordingly.

**Figure 2.**
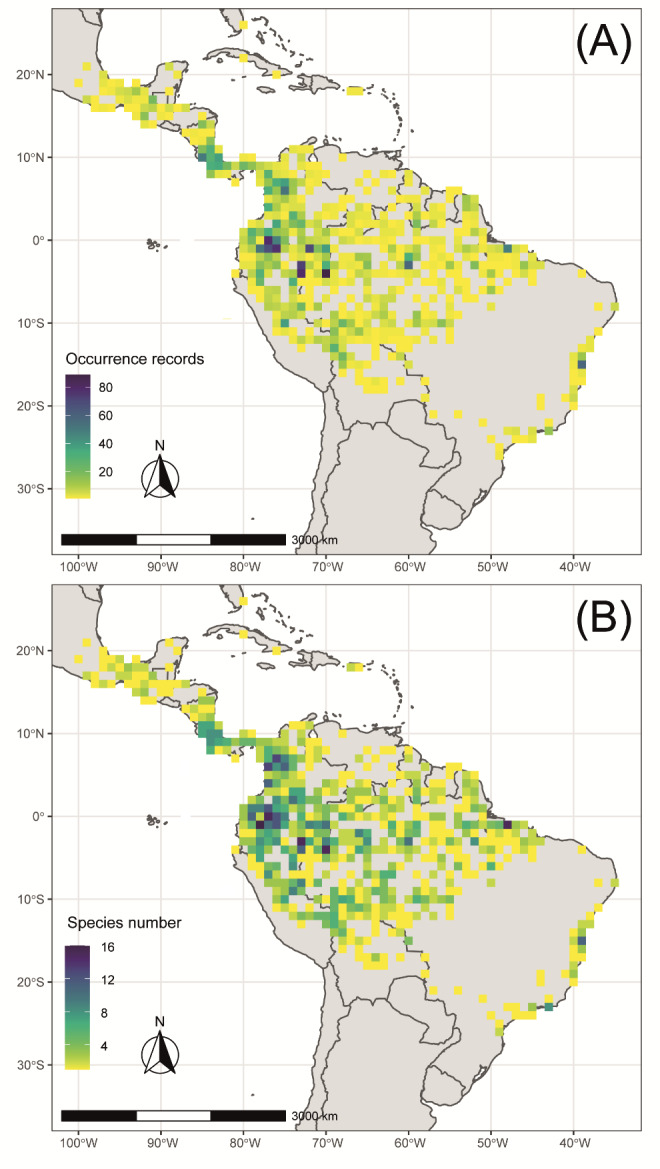
Distribution of preserved specimen occurrences (A) and species richness (B) of cacao and its wild relatives (*Theobroma* and *Herrania*). Tropical Americas at 1º grid-cells. Preliminary results generated on 3 May 2021. Grid maps were made using the “speciesgeocodeR” package v. 2.0 in R Environment (Töpel et al. 2016, R Core Team 2020).

Most *Theobroma* and *Herrania* records are located in Western Amazonia, reaching Panama and Mesoamerica (Fig. 2a,b), which also coincides with regions of species richness in both genera (Fig. 2c,d). Countries with more occurrence records are Brazil (2564 entries, 31% from the total), followed by Colombia (1794 records, 22%), Peru (1094, 13%) and Ecuador (610, 8%). Conversely, countries with more species recorded for the country are Colombia (29 species), Ecuador (23 species), Brazil (18 species), Costa Rica (17 species) and Peru (15 species). For a full relationship of the distribution of all species and records across each country, check Suppl. material 2.

It should be noted that other countries outside the native range of the genera, namely in Africa, Tropical Asia and in the Antilles, are distinguished by having introduced specimens, such as Afghanistan, Trinidad and Tobago and Guinea (see Suppl. material 2).

A few specimens can be found inside Amazonian protected areas or in primary forests along rivers, especially in the region outlined by Colombia, Peru, Ecuador and north-western Brazil. Relevant protected areas with most records are Yasuni National Park, Rio Caquetá, Reserva Faunistica Cuyabeno, Parque Nacional Natural Amacayacu and Parque Nacional Yanachaga-Chemillen. Even though some areas have been extensively collected, some studies even suggest that, in some cases, suitable areas where cacao and relatives occur are mostly unprotected, as seems to be the case for Colombia (González-Orozco et al. 2020).

**Figure 3.**
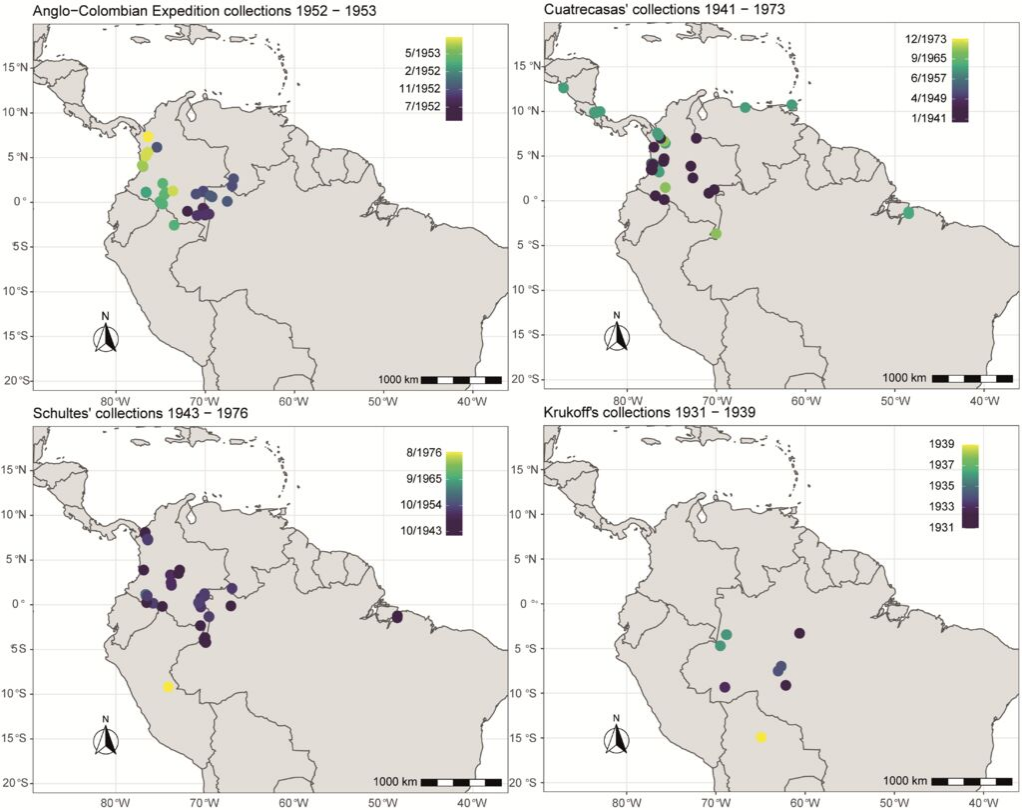
Historical collections of the four selected expeditions of *Theobroma* and *Herrania*, carried out by José Cuatrecasas, Richard E. Schultes, Boris A. Krukoff and the Anglo-Colombian Cacao Collecting Expedition, led by Richard E.D. Baker, Francis William Cope, Paul C. Holliday, Basil G.D. Bartley and D.J. Taylor, from the Imperial College of Tropical Agriculture, Trinidad.

The Anglo-Colombian Cacao Expedition was carried out between 1952 and 1953 by Richard E.D. Baker, Francis William Cope, Paul C. Holliday, Basil G.D. Bartley and D.J. Taylor, with the participation of Richard Schultes, who produced *Herrania*'s monograph (Schultes 1958). The course of this expedition started mostly in eastern Colombia, reaching the north-western limit of Amazonas State, Brazil and southern Venezuela, towards eastern Colombia (Fig. 3). The expedition was an initiative of the Imperial College of Tropical Agriculture of Trinidad and Tobago, led by many botanists interested in wild and cultivated forms of *T.cacao* (Baker et al. 1953). At the time, botanical samples of 13 species of *Theobroma* and 10 species of *Herrania* were made, along with notes on the incidence of witches’ broom that were present in wild cacao specimens.

**Figure 4.**
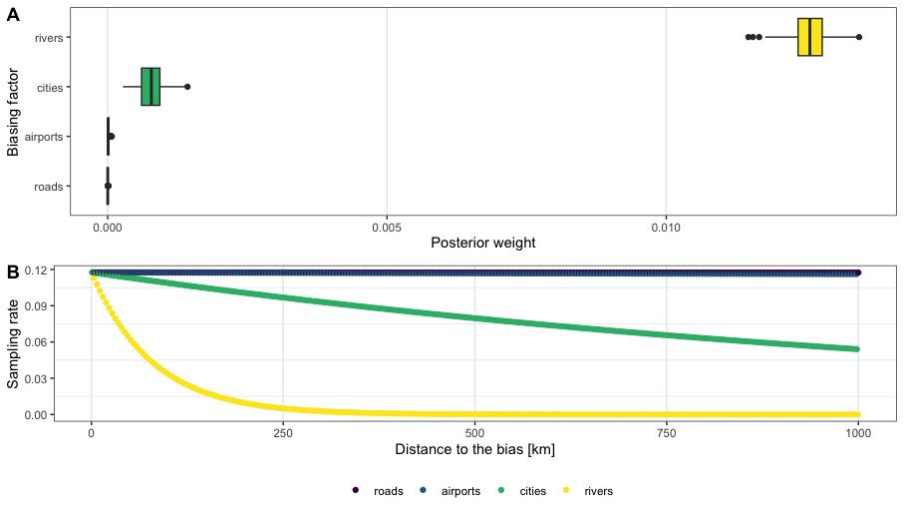
Results of sampling bias analysis, which estimates the effects of the main drivers for collection sampling (collecting near rivers, city areas, airports or roads). At the study scale of 0.25 degrees, "sampbias" found a major relevance of rivers and a moderate relevance of cities in delimiting the collection bias of wild cacao species. Sampling bias analysis was conducted using the package "sampbias" v. 1.0.5 in R Environment (Zizka et al. 2020).

**Figure 5.**
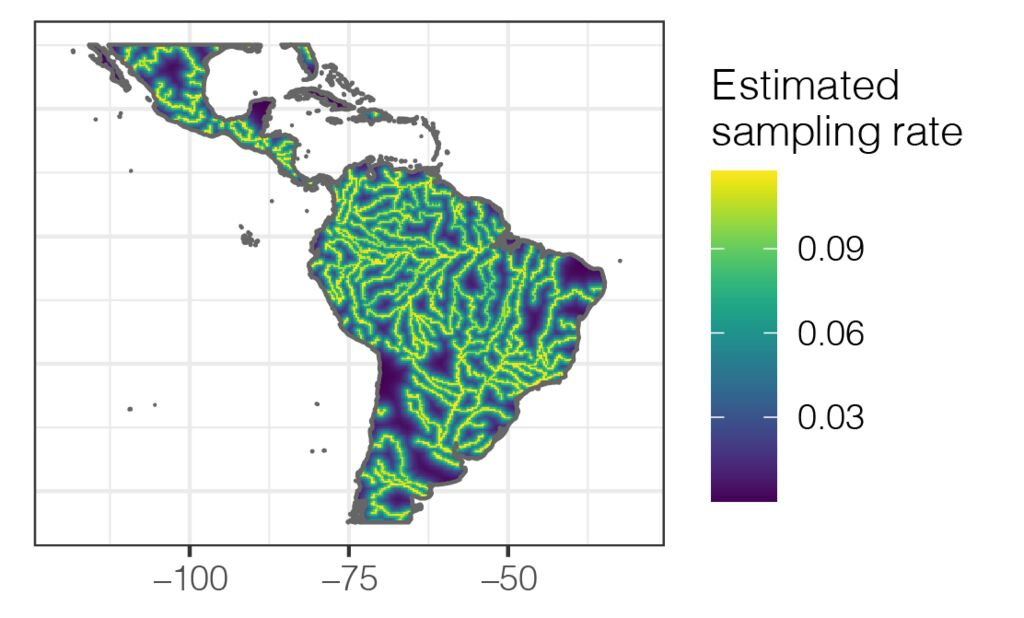
Mapping of sampling bias effects of wild cacao species occurrences in Tropical Americas considering the main drivers for biasing effects (rivers, cities, airports and roads). At the study scale of 0.25 degrees, the mapping shows how river has a major effect in collection biasing for the specimens of this study. Sampling bias mapping analysis was conducted using the package "sampbias" v. 1.0.5 in R Environment (Zizka et al. 2020).

Brazilian Amazonia is relatively less known in collections of *Theobroma* and *Herrania* than other countries, especially considering its larger area. Furthermore, spatial bias in this region is high and most collections are made in areas near rivers or major railways close to urban clusters (Nelson et al. 1990, Vale and Jenkins 2012, Oliveira et al. 2016, ter Steege et al. 2016, Colli-Silva and Pirani 2020). In the case of our study, we found a strong effect of rivers on sampling intensity, followed by a moderate effect of cities (Fig. 4). Colli-Silva and Pirani (2020) highlight a bias for Byttnerioideae (incl. *Theobroma* and *Herrania*), where Amazonian collections are much more biased than collections made in other areas of South America, which agrees with that reported for this study (Fig. 5).

Further collecting endeavours in Brazil, namely the *Projeto Flora Amazônica*, were important for gathering new collections of *Theobroma* and *Herrania* in the Amazon rainforest. The *Projeto Flora Amazônica* took place in the 70s (Prance et al. 1984). Despite being a successful initiative, several areas of the Brazilian Amazonia remain unknown, as can be easily denoted by checking the current numbers of the Brazilian Flora 2020 Project (BFG 2021): although being the largest state in Brazil, Amazonas State is in the fourth position of species-richness of vascular plants, after states, such as Bahia, Minas Gerais and São Paulo States, much smaller in area than Amazonas.

**Figure 6.**
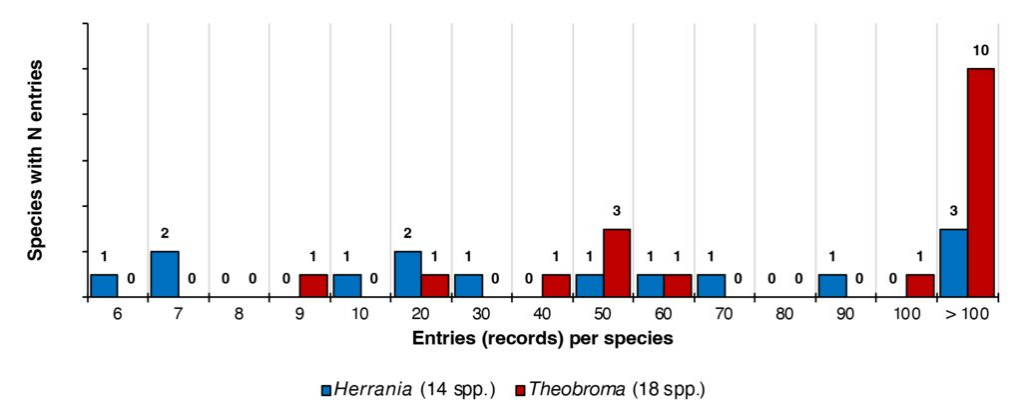
Frequency of occurrence of preserved specimen records of *Theobroma* and *Herrania* species compiled in this study.

Amazonian collections have historically been undocumented and underestimate the real richness of the area (Prance et al. 2000, Schulman et al. 2007, Sousa-Baena et al. 2013, Hopkins 2019). Hopkins 2019 showed that, while most species were collected only in a single event, few species are been collected many times. Interestingly, our results show a shape of the curve that, unlike Hopkins (2019), suggest the prevalence of a documented diversity (Fig. 6), possibly due to considering time efforts of botanical sampling focused on wild cacao species more than other Amazonian groups and also to the fact that many species are found cultivated for crop improvement (Silva et al. 2004). In contrast, Colli-Silva and Pirani (2020) highlight a strong bias effect for both genera in areas of Amazonia, which can reveal areas where there at least should be an increase in the known distribution of the taxon, but where no specimens of the group have been collected.

### Coordinates

-25.591 and 29.644 Latitude; -104.962 and -34.8667 Longitude.

## Temporal coverage

**Data range:** 1760-1-01 – 2020-8-03.

### Notes

**Figure 7.**
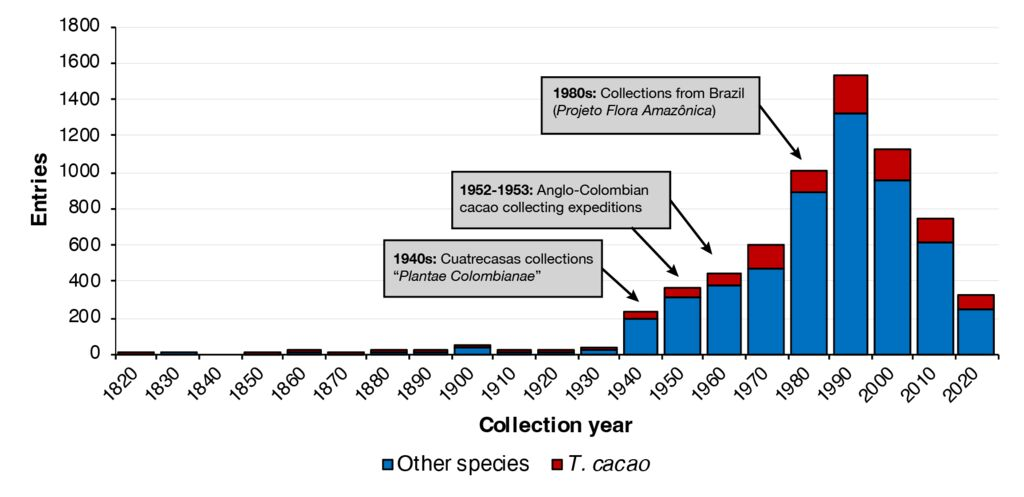
Temporal series of *Theobroma* and *Herrania* collections, highlighting selected major events that influenced the increasing of new collections over decades.

By the time of this analysis, periods of collection peaks are observed in 2014, with 491 new entries in a single year, followed by 1992, with 252 new entries and then by several years from 70s to 90s (Fig. 7).

The history of cacao collecting expeditions is marked by numerous expeditions led by American or European botanists, in contrast with a few led by Latin American teams. Consequently, most preserved specimens are found at American or European herbaria, especially at MO, NY, US, F, U, L and K collections.

Below, we describe a chronological sketch of the most relevant moments where wild cacao species collections were made over the last centuries, according to our dataset and considering the chronology summarised in Fig. 7.*^1^

**ca. 1689**

The epoque of the first known record used as type of a name of *Theobroma*, collected by Sir Hans Sloane (1660-1753), a British physician and naturalist who travelled to the Caribbean, where he documented his travels and collected the first specimen of *Theobromacacao* L. from Jamaica, which was later assigned as the lectotype of *Theobromacacao* L. by Cuatrecasas (1964). The specimen can be found at the London Natural History Museum (BM). Sir Sloane made one of the first descriptions of a popular use of a *Theobroma*, where he was credited as being the first to report the use of *T.cacao* as a bitter drink (Delbourgo 2011).

**1775**

First dated collection made of *Theobroma* with known location and collector. This specimen was collected by Jean Baptiste Aublet (1720-1778), a French botanist who worked with the French Guiana flora. This collection, first labelled as “*Cacaoguianensis* Aubl.”, the type of its name, is originally ascribed to the surroundings Cayenne and it is actually a *Theobromaspeciosum* Mart. The material is deposited at the Natural History Museum (BM).

**1777-1778**

The Spanish botanists Hipólito López (1754-1816) and José Pavón y Jiménez (1754-1840) and the French naturalist Joseph Dombey (1742-1794) led the Botanical Expedition to the Viceroyalty of Peru, collecting more than 3,000 botanical samples deposited mostly in the Royal Botanical Garden of Madrid (MA), with duplicates sent to the Field Museum (F) and to the Missouri Botanical Garden (MO). This expedition culminated in the production of ten volumes of the *Flora Peruviana et Chilensis prodromus* (see Steele (1964)). The type series of *Theobromasinuosum* Pav. ex Huber are some of the important collections from these samples.

**1787-1803**

Accomplishment of “The Spanish Royal Botanical Expedition to New Spain” (*Plantae Novae Hispaniae*), also known as the “[Martín de] Sessé & [José Mariano] Mociño Expedition”, led by many botanists familiar with works of Linnaeus and Nilokaus Jacquin. The expedition was carried out in the actual region of Mexico, Guatemala, Nicaragua, Cuba and Porto Rico reaching the north-western US, with an estimated number of plant collections varying between 8,000-10,000 (McVaugh 2000). Specimens of *T.bicolor* (labelled as *Theobromaovatifolia* Sessé & Mociño, a name not validly published) and *T.cacao*, found cultivated in the area, as well as *T.angustifolium* were collected. Most of these collections are deposited in American herbaria, such as the Field Museum (F) and the Missouri Botanical Garden (MO).

**1825-1830**

William Burchell (1781-1863), an English naturalist, travelled to Brazil collecting a large amount of plants, but especially insects. Such expedition culminated in the publication of *Catalogus Geographics Plantarum Brasiliae Tropicae*. Records of *T.subincanum* and *T.grandiflorum* are part of Burchell’s collections, which can be found in London, at the Royal Botanic Gardens, Kew (K).

**1830**

First known collection of *Herrania* made by Eduard F. Poeppig (1798-1868), a German botanist who worked as a naturalist in Cuba and made expeditions in Chile, Peru and Brazil, publishing *Reise in Chile, Peru und dem Amazonenstrome, während der Jahre 1827-1832*. Collections of *Herranianitida* (Poepp.) R.E.Schult., are from this time. Poeppig’s collections of *Theobroma* are deposited at the Naturalis Biodiversity Center (L, U and WAG collections), Field Museum (F) and at the Natural History Museum of Vienna (W).

**1843-1846**

Justin Goudot (1802-1850), a French naturalist, made field expeditions in Colombia, where he collected many species of vertebrates (Palmer 1918), but also plants, such as *H. albiflora, H. laciniifolia* and *H.pulcherrima*, which comprise the first dated records for these species as well as records that formed the basis for the creation of the genus *Herrania*. Goudot’s duplicates of *Herrania* are deposited at the French National Herbarium (P), Geneva Herbarium (G) and at the Field Museum (F).

**1851**

Richard Spruce (1817-1893), a British botanist, made his first collections of *Theobroma* from this time, with records of *T.sylvestre*, *T.grandiflorum* and *T.speciosum*. These specimens are samples from his journey to Amazonia (dated mostly from 1849 to 1864), starting from the Andes up to the upper Amazon River, collecting in Brazil, Ecuador and Peru (Seaward 2000, Pearson 2004). Most of Spruce’s collections can be found at the Royal Botanic Gardens, Kew (K) and in the New York Botanical Garden (NY).

**1858**

Paul Sagot (1821-1888), a French botanist who collected in Guiana, making new collections of *Theobroma* in the area. Sagot’s collections are deposited at the French National Herbarium (P) and at the Royal Botanic Gardens, Kew (K).

**1874-1875**

James Trail (1851-1919), a Scottish botanist, made expeditions in the Upper Amazon and tributaries, including northern Brazil, where he made collections of *Theobroma*. Trail’s collections are deposited at Royal Botanic Gardens, Kew (K) and at the French National Herbarium (P).

**1880**

Auguste Glaziou (1829-1906), a French botanist, collected in Brazil between 1861 and 1895, making collections of *Theobroma*, which can be found at the French National Herbarium (P).

**1891-1911**

Henry Pittier (1857-1950), a Swiss botanist, explored areas of Panama, Colombia and Venezuela (Dwyer 1973), making several collections of forested areas in these countries, publishing *Primitae Florae Costaricensis* and *Herborisations au Costa Rica* and depositing most materials at the Smithsonian National Herbarium (US), French National Herbarium (P), Field Museum (F), Royal Botanic Gardens, Kew (K) and at the National Museum of Costa Rica (CR).

**1904-1969**

Adolpho Ducke (1876-1959), an Austrian botanist naturalised in Brazil, made several collections in the Brazilian Amazon, where he studied many plants and published several works for the area, including with *Theobroma* (Ducke 1940). Most of Ducke's collections can be found at the Emilio Goeldi Museum in Belém, Brazil (MG).

**1905-1919**

Auguste Chevalier (1873-1956), a French botanist, made new collections of *Theobroma* species, especially *T.cacao* from Africa, where he studied *T.cacao* morphotypes and cacao cultivar classification.

**1914**

Orator Cook (1867-1949) and Conrad Doyle (1884-1973), both American botanists from the Smithsonian Institution (US), led expeditions in Mexico, Colombia, Costa Rica and Guatemala, where they identified stilt palms and collected, amongst other species, cacaos from Guatemala.

**1903-1910**

A team of Dutch botanists arrived in Suriname, collecting specimens of *Herrania* from the area which, after World War II, were all sent to the Naturalis Biodiversity Center collection of Utrecht (U) (Klooster et al. 2003).

**1906-1929**

Walter Broadway (1863-1935), an English naturalist, served as gardener in the Royal Botanic Gardens (K) and later as superintendent in Trinidad, where he made *Theobroma* collections also in French Guiana and Venezuela. Most of his duplicates are found in BM, K, MO and P.

**1929-1942**

Llewelyn Williams (1901-1980), an English botanist who was interested in botanical products from tropical regions, conducted extensive field expeditions in northern South America, following the margins of the Orinoco River Basins. Most of his collections are deposited at the Field Museum (F).

**1916-1948**

Ellsworth Killip (1890-1968) and Albert Smith (1906-1999), American botanists from the Smithsonian Institution (MO), collected extensively in Colombia, Brazil, Cuba, Jamaica, Panama, Peru and Venezuela, where they had the opportunity to collect wild cacao species from these areas. Duplicates were mostly sent to MO, F and US.

**1920-1933**

Guillermo Klug (-1946), a Peruvian parabotanist, made extensive collections in Amazonian Peru and Colombia, contributing with the knowledge of wild cacao species and other elements of the flora of the area. Most of its specimens and notes were sent to US herbaria, with duplicates at F and NY.

**1928-1950**

Boris A. Krukoff (1898-1983), a Russian botanist, led numerous expeditions in Amazonia, collecting wild cacao species mostly between 1931 and 1939 in the Basin of Rio Solimões in Brazil.

**1938-1945**

Frederick J. Pound (1919-1944), a British biologist from the Imperial College Station of London, established the first cacao germplasm collection, leading expeditions in Upper Amazonia, in Rio Ucayali, Rio Morona and Rio Marañón in Peru and Ecuador (Zhang et al. 2009) to find new cultivars of cacao, collecting pods from trees. Most specimens were not deposited in herbaria and are kept only as germplasm.

**1939-1969**

José Cuatrecasas (1903-1996), a Spanish botanist from the Jardim Botánico de Madrid (MA), conducted extensive trips in South America, collecting in Colombia, Venezuela and Ecuador. Cuatrecasas spent years of his life studying plants, with a particular focus in the genus *Theobroma*, describing new species and publishing the seminal taxonomic revision of the genus (Cuatrecasas 1964). Most of Cuatrecasas’s collections are found at the Smithsonian Institution (US).

**1942-1960**

Richard E. Schultes (1915-2001), an American ethnobotanist from Harvard University, led expeditions in South America and Mexico, mostly looking for useful plants used by indigenous people. During this time, he also became interested in the wild cacao species, especially those of the genus *Herrania*. His interest and fieldwork resulted in the publication of *Herrania*’s synopsis (Schultes 1958), a gold standard for the taxonomy of the genus. Most of his collections are found in American herbaria, namely US, F, GH and MO.

**1942**

William Archer (1894-1973), an American economic botanist from the Smithsonian Institution (US), carried out expeditions in Pará, Brazil, where he collected many samples of *Theobroma*. Most of the duplicates were sent to US and F.

**1945-1946**

Ricardo Fróes (1891-1960), a Brazilian botanist associated to the Instituto Agronômico do Norte, in Belém do Pará, led expeditions in the region of Fonte Bôa, Amazonas, Brazil, from where some collections of *Theobroma* are derived.

**1953-1967**

Elbert Luther Little, Jr. (1907-2004) and Ruby Rema Little (1907-2009), both American botanists, collected in Venezuela and Costa Rica. Most duplicates of these expeditions can be found at F.

**1951-1963**

Victor Patiño (1912-2001), a Colombian botanist, led expeditions in Andean countries (Venezuela, Colombia, Peru, Ecuador, Bolivia and Chile), depositing most of his samples at Medellín Germplasm Bank with duplicates sent to F and US collections.

**1952-1953**

Period of the Anglo-Colombian Cacao Collecting Expedition. With expeditions led by the American botanists in collaboration with the Imperial College of Tropical Agriculture of Trinidad and the Colombian Government, the areas explored included the rivers Caquetá, Apaporis, Vaupés, Negro and tributaries towards Putumayo and El Chocó (Baker et al. 1953), collecting almost 200 botanical samples, mostly of *T.cacao*, but other species of *Theobroma* and *Herrania*. The Anglo-Colombian Cacao Collecting Expedition counted with the interaction of Schultes and Cuatrecasas. Many specimens from these expeditions are found in American collections, especially F and US, but also at COL in Bogotá, Colombia.

**1963-1975**

Roelof Oldeman (1937-), a Dutch botanist from the Natural History Museum (BM), made several trips to the Guianas and northern Brazil, collecting samples of *Theobroma* and *Herrania*. Most of its wild cacao species collections can be found at U, US and P.

**1965-1966**

Basett Maguire (1904-1991), an American botanist from the New York Botanical Garden (NY), led an expedition to the Serra da Neblina Expedition, collecting in the region of Rio Negro and Rio Cauaburí, in Brazil. This expedition was conducted by the University of Brasilia in conjunction with the Instituto Nacional de Pesquisas da Amazonia (INPA) and the New York Botanical Garden (NY), with funds from the National Science Foundation. Maguire's collections from that time can be found at INPA and NY.

**1964-1989**

Ghillean T. Prance (1937-), an English botanist, led the *Projeto Flora Amazônica*, an initiative funded by the Brazilian Government and the National Science Foundation, aiming at collecting in particular areas of the Brazilian Amazonia. Collections from this project include *Theobroma* and *Herrania* and are mainly found at INPA, US and NY.

**1968-1972**

Thomas Croat (1938-), an American botanist interested in systematics and ecology of Araceae, made expeditions in the region of Loreto, in Peru, where he collected samples of wild *Theobroma* and *Herrania* species, mostly deposited at F, MO and NY.

**1969-2005**

José Schunke-Vigo (1929-2018), a Peruvian botanist, collected *Theobroma* and *Herrania* especially in the Peruvian Amazonia, contributing greatly with the Flora of Peru (Croat and Graham 2019). Most of his specimens were deposited at F and US.

**1971-1991**

Paul Maas (1939-), a Dutch botanist from Urecht University (U), carried out expeditions in the Guianas and in Ecuador to publish floristic treatments for these regions, where he also collected *Theobroma* and *Herrania*. Maas travelled to over twenty countries, often visiting each place more than once and he was mostly accompanied by other colleagues and students on his trips (Koek-Noorman 2004).

**1973-1983**

Ronald Liesner (1944-), an American Botanist from the Missouri Botanical Garden (MO), made expeditions in the region of Costa Rica and Panama, collecting samples of *Theobroma* and *Herraniapurpurea*, with most materials found at MO.

**1976-1986**

Juan Revilla, a Peruvian botanist working in the Instituto Nacional de Pesquisas da Amazônia (INPA), Brazil, led expeditions in Peru, mostly under the auspices of the Flora do Peru project, in collaboration with the Missouri Botanical Garden (MO) and the Field Museum (F), funded by the National Science Foundation. Most of Revilla's collections can be found at F, INPA and MO.

**1974-1997**

Scott Mori (1941-2020), an American botanist from the New York Botanical Garden (NY), coordinated expeditions in several sites of Brazil and Suriname, the latter supported by the Fund for Neotropical Plant Research. Most of Mori's *Theobroma* and *Herrania* samples were sent to American collections of US and NY.

**1976-1978**

The Project “Plantas da Amazônia”, also funded by the National Science Foundation in conjunction with Brazilian Government, explored areas Brazil’s Amapá State, with most *Theobroma* and *Herrnia* samples found at MO, F and US.

**1980-1986**

Carlos D. Cid-Ferreira, a Brazilian botanist, based at the Instituto Nacional de Pesquisas da Amazonia, led several expeditions to different areas of Amazonia, including Acre, Rondônia, Pará and Amazonas States, reaching newly-collected areas. Many vouchers of *Theobroma* and *Herrania* collected in this occasion were deposited at INPA and duplicates were sent to American collections.

**1989-1999**

Marion Jansen-Jacobs (1944-), a Dutch botanist, made expeditions in the Guianas, in association with the Utrecht University (U), where most of his samples of *Theobroma* and *Herrania* species can be found.

**2000-onwards**

Collections of different authors prevailed from that time and focused expeditions became less recurrent. In fact, many of the recent expeditions are characterised by revisiting recollected spots. One exception is the Colombian Expedition "Cacao BIO" conducted in 2020, where more than 5000 samples and 200 samples of wild cacao species were collected in many parts of Colombia. This expedition was coordinated by the Corporación Colombiana de Investigación Agropecuaria - AGROSAVIA and the dataset is avaialble in GBIF (González-Orozco et al. 2021). Although our study did not consider the dataset from Cacao BIO, because the entries did not consist of preserved specimen occurrences, Cacao BIO is a remarkable expedition in terms of newly-collected samples and one of the largest made so far, at least for Tropical Americas, in terms of biological sampling.

Four botanical expeditions are relevant to the increase of wild cacao species collections, as described in Fig. 3: (1) the Anglo-Colombian Cacao Expedition collection, (2) expeditions made by José Cuatrecasas and (3) Richard E. Schultes and (4) Boris A. Krukoff collections in Brazil.

## Usage licence

### Usage licence

Other

### IP rights notes

Attribution 4.0 International (CC BY 4.0).

## Data resources

### Data package title

GBIF Occurrence Download 10.15468/dl.yze9k4

### Resource link

https://doi.org/10.15468/dl.yze9k4

### Alternative identifiers

0032886-200613084148143

### Number of data sets

2

### Data set 1.

#### Data set name

GBIF Occurrence Database 10.15468/dl.yze9k4

#### Data format

List

#### Download URL

https://doi.org/10.15468/dl.yze9k4

#### Data format version

1.0

#### Description

GBIF Occurrence Dataset, with 15,849 occurrences included in download.

**Data set 1.** 

Column label	Column description
citations.txt	Provide citations to the datasets consulted to merge the dataset.
meta.xml	Specify the structure of the occurrence.txt file.
metadata.xml	Specify the structure of the whole dataset.
multimedia.txt	Disposes the links to access image files for entries with digitised vouchers or entries with photos associated.
occurrence.txt	Provides the occurrence dataset in DarwinCode format.
rights.txt	Lists the right licence for all datasets used in this dataset.
verbatim.txt	Provides the occurrence dataset in DarwinCode format.
dataset	Folder containing metafiles for all datasets used in this database.

### Data set 2.

#### Data set name

Final dataset used for this work, based on GBIF Occurrence Datasets

#### Data format

DarwinCore plus additional fields

#### Description

Dataset resultant from GBIF-mobilised data, after curation, cleaning, georeferencing and selection of wild preserved specimen collections of *Theobroma* and *Herrania* from Tropical Americas and overseas.

**Data set 2.** 

Column label	Column description
basisOfRecord	The specific nature of the data record.
gbifID	Unique identifier for an occurrence record in GBIF.
taxonRank	The taxonomic rank of the most specific name in the scientificName.
genus	The full scientific name of the genus in which the taxon is classified.
scientificName_after_revision	The full scientific name, with authorship, after manual revision of the record.
scientiticName_original	The full scientific name, with authorship, as originally informed in the dataset prior revision.
decimalLatitude_after_revision	The geographic latitude (in decimal degrees) of the geographic centre of a Location, after manual revision and georeferencing.
decimalLongitude_after_revision	The geographic longitude (in decimal degrees) of the geographic centre of a Location, after manual revision and georeferencing.
licence	A legal document giving official permission to do something with the resource.
institutionCode	The name (or acronym) in use by the institution having custody of the object(s) or information referred to in the record.
collectionCode	The name, acronym, coden or initialism identifying the collection or dataset from which the record was derived.
datasetName	The name identifying the dataset from which the record was derived.
ownerInstitutionCode	The name (or acronym) in use by the institution having ownership of the object(s) or information referred to in the record.
catalogNumber	An identifier (preferably unique) for the record within the dataset or collection.
recordedBy.new	Name of the primary collector for recording the original occurrence, after data standardisation.
recordNumber.new	Collection number for recording the original occurrence, after data standardisation.
recordedBy	Name of the primary collector for recording the original occurrence, as originally informed in the record, prior standardisation.
recordNumber	Collection number for recording the original occurrence, as originally informed in the record, prior to standardisation.
eventDate	The date-time or interval during which an Event occurred (ISO 8601-1:2019).
countryCode	The standard code for the country in which the Location occurs (ISO 3166-1-alpha-2), as originally informed in the record, prior to revision.
stateProvince	The name of the first administrative region (state, province, canton, department, region etc.) in which the Location occurs, as originally informed in the record, prior to revision.
county	The full, unabbreviated name of the second administrative region (county, shire, department etc.) in which the Location occurs, as originally informed in the record, prior to revision.
municipality	The full, unabbreviated name of the third administrative region (city, municipality etc.) in which the Location occurs, as originally informed in the record, prior to revision.
locality	Less specific geographic information can be provided in other geographic terms (higherGeography, continent, country, stateProvince, county, municipality, waterBody, island, islandGroup), as originally informed in the record, prior to revision.
imageChecking	Image checking criteria after assessing the record for revision, categorised as "No image seen to examine voucher, look at herbaria", "Not seen at herbaria, but image seen online properly", "Physically seen at herbaria and checked at herbarium" or "Voucher not seen online, but image of one or more of its duplicates seen".
georeferencingChecking	Georeferencing checking after assessing the record information on geographic occurrence, categorised as "Coordinates previously informed dubious or ambiguous and could not correct properly", "Coordinates previously informed in the label and not altered", "Could not georeference properly" or "Georeferencing corrected accordingly".
country.new	The full name of country or territory in which the Location occurs, after occurrence revision.
stateProvince.new	stateProvince in which the Location occurs, after occurrence revision.
municipality.new	Municipality in which the Location occurs, after occurrence revision.
locality.new	Locality in which the Location occurs, after occurrence revision.
Resol.orig	Resolution of the occurrence record prior to data revision.
Resolution.stand	Resolution of the occurrence record after data revision.
loc.check	Occurrence transformation status after standardisation.

## Supplementary Material

E08A3BD4-EABD-539B-9064-FFF9921B1CBD 10.3897/BDJ.11.e99646.suppl1 Supplementary material 1 Revisited dataset of biodiversity data of wild entries of *Theobroma* and *Herrania* (Malvaceae, Byttnerioideae) from Tropical Americas. Data type Preserved specimen occurrences Brief description Species occurrence dataset, with preserved specimen records of species of *Theobroma* and *Herrania*, after downloading the preliminary dataset from GBIF and providing the data manipulation framework. File: oo_789005.txt https://binary.pensoft.net/file/789005 Matheus Colli-Silva; James Edward Richardson; José Rubens Pirani

D408B520-3AEF-51C6-B54C-645651837566 10.3897/BDJ.11.e99646.suppl2 Supplementary material 2 Full relationship of record distribution of *Theobroma* and *Herrania* across countries in Tropical Americas and overseas Data type Distribution data Brief description Full description of the preserved specimen collection records across each country in Tropical Americas, per species of *Theobroma* and *Herrania*. File: oo_789006.txt https://binary.pensoft.net/file/789006 Matheus Colli-Silva; James Edward Richardson; José Rubens Pirani

## Figures and Tables

**Table 1.** Classes of georeferenced data according to coordinate revision. Based on data of Suppl. material 1.

**Checking status**	**Entries**	**Percent**
Coordinates maintained or assigned according to the information on the label	5277	66%
Previously informed coordinates dubious or ambiguous and could not be properly corrected	1960	25%
Georeferencing corrected accordingly	738	9%
**All entries**	**7975**	**100**%

## Footnotes

*1 Much of the biographic data of authors were taken from Brummitt and Powell (1992), unless explicitly mentioned. Herbarium acronyms follow Thiers (2021).
